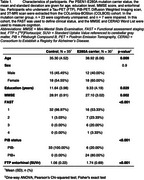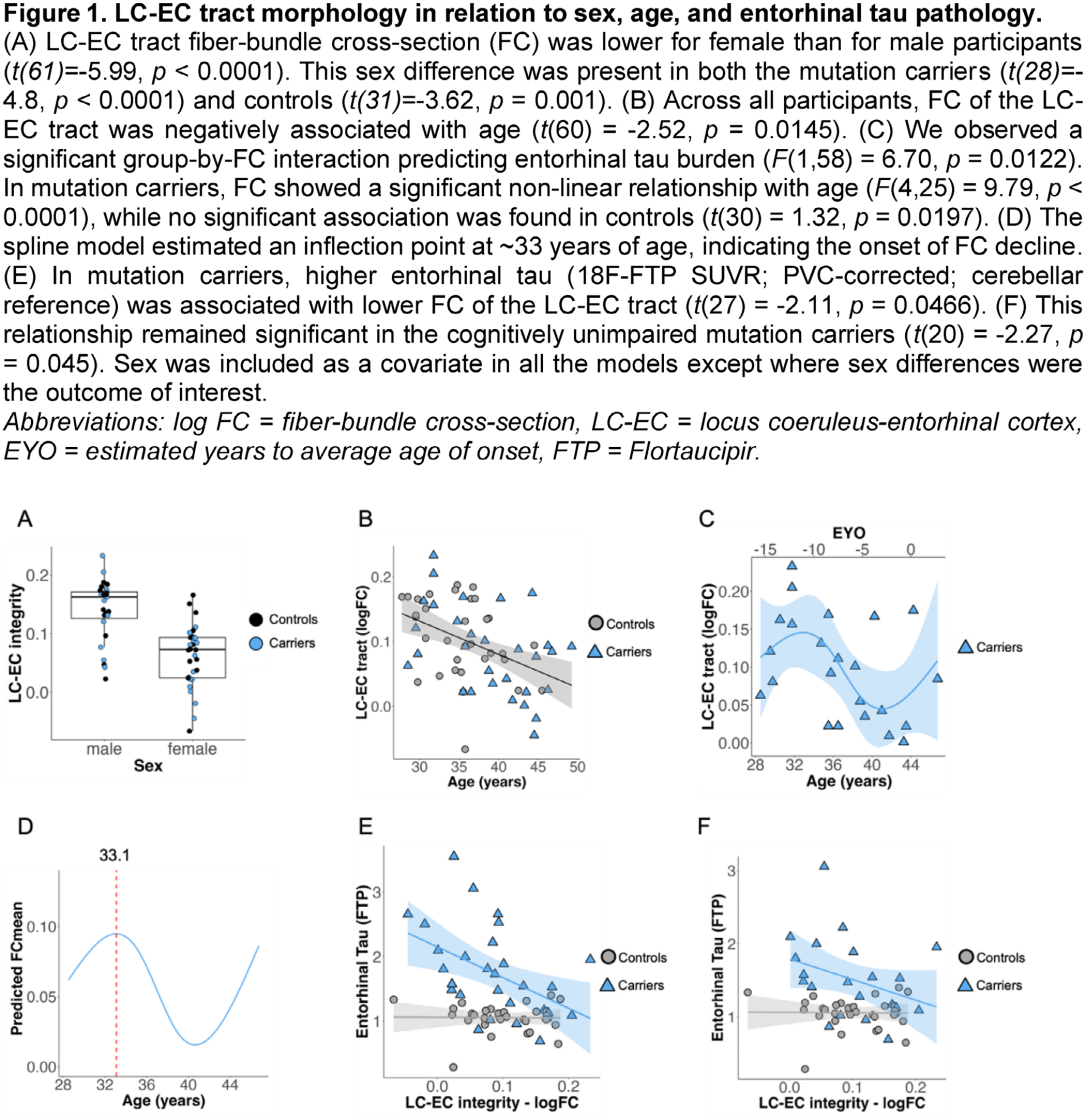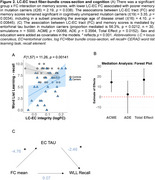# LC‐EC Tract Degeneration is associated with Entorhinal Tau and Memory in Autosomal‐dominant Alzheimer’s Disease

**DOI:** 10.1002/alz70861_108445

**Published:** 2025-12-23

**Authors:** Elouise A. Koops, Averi Giudicessi, Ana Y Baena, Nikole A Bonillas Félix, Randy Medrano, Lusiana Martinez, Isabela Gonzalez, Sergio Alvarez, David Aguillon, Yakeel T. Quiroz, Heidi I.L. Jacobs

**Affiliations:** ^1^ Athinoula A. Martinos Center for Biomedical Imaging, Massachusetts General Hospital, Harvard Medical School, Boston, MA USA; ^2^ Massachusetts General Hospital, Harvard Medical School, Boston, MA USA; ^3^ Grupo de Neurociencias de Antioquia, Facultad de Medicina, Universidad de Antioquia, Medellín Colombia; ^4^ Hospital Pablo Tobón Uribe, Medellín, Antioquia Colombia; ^5^ Grupo de Neurociencias de Antioquia, Facultad de Medicina, Universidad de Antioquia, Medellín, Antioquia Colombia; ^6^ Boston University, Boston, MA USA; ^7^ Grupo de Neurociencias de Antioquia, University of Antioquia, Colombia, Medellín, Antioquia Colombia; ^8^ Faculty of Health, Medicine and Life Sciences, School for Mental Health and Neuroscience, Alzheimer Centre Limburg, Maastricht University, Maastricht Netherlands

## Abstract

**Background:**

The locus coeruleus (LC) is among the earliest regions accumulating tau in sporadic and autosomal dominant Alzheimer’s disease (AD‐AD), preceding tau in the entorhinal cortex (EC). A proposed mechanism of tau progression is along LC projections, contributing to LC‐EC tract degeneration. Here we examined LC‐EC tract morphology in the context of disease progression in AD‐AD.

**Method:**

We examined LC‐EC white matter tract characteristics derived from diffusion‐weighted images in PSEN1‐E280A mutation carriers (*n* =30) and controls (*n* =33) from the Colombia‐Boston cohort using fixel‐based analysis and tractography. Clinical status was defined using the FAST, and the MMSE and CERAD Word List assessed cognition. Linear regressions tested associations between LC‐EC tract characteristics and estimated‐years‐to‐onset, age, sex, entorhinal tau (18F‐FTP PET), and memory. A mediation analysis assessed the role of entorhinal tau in linking LC‐EC structure to cognition.

**Result:**

LC‐EC tract fiber cross‐section (FC) was lower in females than males (Figure 1A) and declined with proximity to symptom onset in mutation carriers (Figure 1B,C), starting around age 33 (Figure 1D) in cognitively unimpaired individuals. In mutation carriers, lower FC was associated with higher entorhinal tau (Figure 1E,F) and poorer memory (Figure 2A). The relationship between FC and cognition was mediated by tau (56.3% mediated; Figure 2B,C).

**Conclusion:**

We observed LC‐EC tract degeneration in cognitively unimpaired mutation carriers, particularly in females. This degeneration was associated with higher entorhinal tau and poorer cognition, starting several years before symptom onset. LC‐EC tract degeneration temporally aligns with previously reported emerging memory decline, highlighting it as a potential early disease vulnerability marker.